# Spontaneous intracerebral hemorrhage – patients retrospectively consent to fibrinolytic surgery despite poor neurological outcome and reduced health-related quality of life

**DOI:** 10.1007/s10143-024-02479-w

**Published:** 2024-06-12

**Authors:** Regina Schwiddessen, Vesna Malinova, Nicole von Steinbüchel, Dorothee Mielke, Veit Rohde, Christian von der Brelie

**Affiliations:** 1 Department of Neurosurgery, University Medical Center Göttingen, Robert-Koch-Straße 40, 37075 Göttingen, Göttingen, Germany; 2 Department of Medical Psychology and Medical Sociology, University Medical Center Göttingen, Göttingen, Germany; 3 Department of Neurosurgery and Spine Surgery, Johanniter-Kliniken Bonn, Bonn, Germany; 4 Institute of Psychology, University of Innsbruck, Innsbruck, Austria; 5 Department of Neurosurgery, University Medical Center Augsburg, Augsburg, Germany

**Keywords:** Intracerebral hemorrhage, Stroke, Fibrinolytic therapy, Health-related quality of life, Outcome

## Abstract

Spontaneous intracerebral hemorrhage (ICH) might lead to devastating consequences. Nonetheless, subjective interpretation of life circumstances might vary. Recent data from ischemic stroke patients show that there might be a paradox between clinically rated neurological outcome and self-reported satisfaction with quality of life. Our hypothesis was that minimally invasive surgically treated ICH patients would still give their consent to stereotactic fibrinolysis despite experiencing relatively poor neurological outcome. In order to better understand the patients’ perspective and to enhance insight beyond functional outcome, this is the first study assessing disease-specific health-related quality of life (hrQoL) in ICH after fibrinolytic therapy. We conducted a retrospective analysis of patients with spontaneous ICH treated minimally invasive by stereotactic fibrinolysis. Subsequently, using standardized telephone interviews, we evaluated functional outcome with the modified Rankin Scale (mRS), health-related Quality of Life with the Quality of life after Brain Injury Overall scale (QOLIBRI-OS), and assessed retrospectively if the patients would have given their consent to the treatment. To verify the primary hypothesis that fibrinolytic treated ICH patients would still retrospectively consent to fibrinolytic therapy despite a relatively poor neurological outcome, we conducted a chi-square test to compare good versus poor outcome (mRS) between consenters and non-consenters. To investigate the association between hrQoL (QOLIBRI-OS) and consent, we conducted a Mann-Whitney U-test. Moreover, we did a Spearman correlation to investigate the correlation between functional outcome (mRS) and hrQoL (QOLIBRI-OS). The analysis comprised 63 data sets (35 men, mean age: 66.9 ± 11.8 years, median Hemphill score: 3 [2-3]). Good neurological outcome (mRS 0–3) was achieved in 52% (33/63) of the patients. Patients would have given their consent to surgery retrospectively in 89.7% (52/58). These 52 consenting patients comprised all 33 patients (100%) who achieved good functional outcome and 19 of the 25 patients (76%) who achieved poor neurological outcome (mRS 4–6). The mean QOLIBRI-OS value was 49.55 ± 27.75. A significant association between hrQoL and retrospective consent was found (*p* = 0.004). This study supports fibrinolytic treatment of ICH even in cases when poor neurological outcome would have to be assumed since subjective perception of deficits could be in contrast with the objectively measured neurological outcome. HrQoL serves as a criterion for success of rtPa lysis therapy in ICH.

## Introduction

Despite various therapeutic options, intracerebral hemorrhage (ICH) is associated with a high mortality rate up to 50% within the first 30 days and up to 60% in the first year [1, 2]. Moreover, only 12–39% of survivors of ICH achieve long-term functional independence [1]. Regarding the evaluation of therapeutic success, the focus lies on the improvement of functional outcome and the reduction of mortality. However, various studies have shown a significant benefit of surgical treatment options compared to conservative therapy [3, 4]. For example, comparing minimally-invasive fibrinolytic therapy with standard medical care, the MISTIE-III trial demonstrated a significantly lower mortality rate, but it failed to show significant improvement in functional outcome [5]. Consequently, treatment recommendations for ICH are controversial. Furthermore, the reduction of the mortality rate leads to a higher rate of individuals with disabilities that may persist for years. Thus, in evaluating therapy success, the focus should shift more to the assessment of the individual acceptance of disabilities by asking for e.g. retrospective consent and by evaluating health-related quality of life (hrQoL). The question of retrospective consent somehow implies that neurologically impaired patients are satisfied with their subjective life circumstances [6]. A patient-reported outcome measure as hrQoL allows the assessment of a patient’s subjective health status and wellbeing [7]. This construct comprises functional capacity, as well as physical, psychological (emotional, cognitive), social, and everyday well-being, which may be related to or influenced by the presence of a disease or treatment [8–10]. The Quality of life after traumatic brain Injury Overall scale (QOLIBRI-OS) is a valid and reliable instrument for the assessment of disease-specific hrQoL after brain injury e.g. ischemic stroke patients [11]. Currently there is no data on disease-specific hrQoL and the individual acceptance of the life circumstances after fibrinolytic treated hemorrhagic stroke. Thus the influence of this therapy on subjective perceptions of hrQoL and the subsequent conditions is yet unclear. Thus, the aim of this study is to evaluate these two relevant outcome variables. The hypothesis, that fibrinolytic treated ICH patients would still retrospectively consent to fibrinolytic therapy despite a relatively poor neurological outcome, is examined.

## Materials and methods

### Patient population and clinical setting

The present study is in part a retrospective cross-sectional study, which is supplemented by a prospective data collection. Ethical approval (3/11/20) was granted by the local IRB. The guidelines of the current version of the Declaration of Helsinki were followed [12]. All patients suffering from intracerebral hemorrhage (ICH) between January 1st 2010 and October 4th 2020 who were treated with fibrinolytic therapy at the Department of Neurosurgery at the University Medical Center Göttingen were selected for the retrospective data analysis. All patients were treated < 72 h after diagnosis. Patients who were discharged alive received an information letter in advance to the phone interview. These patients classified for further analysis.

### Data collection, inclusion and exclusion criteria

Patient-related and clinical data were retrospectively extracted. The ICH score was used [13, 14]. Only patients with supratentorial ICH are eligible for fibrinolytic therapy, thus patients with infratentorial ICH were excluded. For general eligibility for fibrinolytic therapy, patients had to fulfill the following requirements: The hematoma volume had to be between 30 ml and 150 ml and the GCS had to be between 6 and 12 points prior to intubation. Patients with bilateral fixed and dilated pupils were deemed to have a fatal prognosis and were not suitable for MIS treatment. Patients with secondary ICH caused by a hemorrhagic infarction or trauma were also not suitable. The final decision for or against fibrinolytic therapy was subject to the judgment of the neurosurgeon. This is the reason why some patients received the therapy even though they were not in the above-mentioned ranges in terms of GCS and volume. The functional independence at discharge was measured by modified Rankin Scale (mRS) [15]. Race/ethnicity-based differences were not present.

### Instruments

For the prospective data collection, a questionnaire was developed including different components as a question about retrospective consent for ICH treatment. Long-term functional outcome was measured by the modified Rankin Scale (mRS), which serves as a reliable score for evaluation also on phone interviews by patients or proxies [16]. A dichotomization into favorable (mRS ≤ 3) and unfavorable outcome (mRS 4–6) was performed [5, 17]. As a valid and reliable instrument to assess disease-specific hrQoL, the Quality of life after traumatic brain Injury Overall scale (QOLIBRI-OS) was applied [11, 18]. This tool captures satisfaction with different life dimensions during the previous week on a Likert scale from 0 to 5 (“not at all” “somewhat,” “moderately,” “fairly,” and “very”). The scores for each item were then added together and divided by the number of responses given to obtain the mean score. Scores were then transformed to a scale from 0 (worst) to 100 (best quality of life) [18]. Additionally, the sociodemographic status, which included marital status, occupational status, living situation, each before and after ICH, the highest level of education and the existence of own children was evaluated.

### Procedure

First, potential participants received an information letter, the consent form, and a template of the interview questions. A minimum of 7 months elapsed between ICH and contact. This minimized the discrepancy of assessment between proxy and patient [19]. The interview was only conducted after the consent form was returned. In order to adequately address patients who were not able to return the letter or sign the consent, those patients were called two weeks after the letters were sent. They were included after they consented their participation. A standardized telephone interview was performed. If a patient was unable to participate in person, the assessment was based on relatives’ or primary caregivers’ ratings. The interviews were carried out by a single interviewer (R.S.) minimizing the risk of potential bias from multiple interviewers. The interviewer R.S. is Regina Schwiddessen, a PHD student and the first author of this manuscript. There was no blinding to the treatment during the interview. If it was not possible to make contact either by post or telephone, the status of the patient (living/deceased) was requested from the relevant residents’ registration offices. The documentation was pseudonymized.

### Statistical analysis

All statistical analyses were conducted using IBM SPSS software version 28.0. A descriptive analysis was performed by reporting frequencies, medians, means and standard deviations. Quantitative variables were tested for normal distribution by Kolmogorov-Smirnov test (*p* < 0.05). The Wilcoxon test was used for dependent and the Mann-Whitney U-test for independent ordinal, discrete and continuous variables. Thus, the Mann-Whitney U-test was conducted to investigate the association between hrQoL (QOLIBRI-OS) and consent. Differences in variances between two groups were calculated using chi-square test or Fisher exact test when appropriate. To verify the primary hypothesis that fibrinolytic treated ICH patients would still retrospectively consent to fibrinolytic therapy despite a relatively poor neurological outcome (mRS 4–6), these tests are used. Spearman correlation was chosen for the calculation of correlations, for example to investigate the correlation between functional outcome (mRS) and hrQoL (QOLIBRI-OS). A p-value ≤ 0.05 was considered statistically significant when interpreting the results by using two-sided tests. Missing data were addressed as missing at random (MAR) and subjected to pairwise deletion.

## Results

### Patient characteristics

312 patients with ICH underwent fibrinolytic therapy in the Department of Neurosurgery at the University Medical Center Göttingen. 98 patients (mortality rate = 31.5%) died in the hospital, 10 patients were assigned for palliation. Consequently, the potential collective for follow-up-assessment consisted of 204 patients. Of these, 80 were still alive when the prospective data collection was conducted, 100 patients died during the follow-up period, no information was available from another 24 patients. The final prospective collective consisted of 63 affected individuals. Twenty-eight females and 35 males with a mean age of 66.9 ± 11.8 years at diagnosis were included. Most participants had an ICH score of three [2-3] (shown in Table 1).

**Table 1:** Clinical and demographic parameters of all potential participants, divided into follow-up participants [*n* = 63] and non-participants [*n* = 141]

Parameters	Participants *n* = 63	Non-participants *n* = 141	*p*-value
Female	28 (44.4%)	67 (47.5%)	0.762
Age (years)	66.9 ± 11.8	68.4 ± 13.0	0.237
Anticoagulation	16 (26.7%) [*n* = 60]	40 (28.8%) [*n* = 139]	0.442
Antiplatelet	12 (20.0%) [*n* = 60]	35 (25.2%) [*n* = 139]	0.266
Arterial hypertension	37 (58.7%)	84 (60.4%) [*n* = 139]	0.877
Gcs†	11 [10-12]	11 [8-12] [*n* = 136]	0.087
Ich‡ score	3 [2-3]	3 [2-3] [*n* = 136]	0.555
Baseline ICH volumen (ml)	47.4 ± 21.3	49 ± 22.1	0.640
Deep ICH	29 (46.0%)	70 (49.6%)	0.652
Intraventricular hemorrhage	43 (68.3%)	89 (63.1%)	0.528
Days at hospital	21.3 ± 9.5	21.3 ± 11.3	0.741
Days at intensive care unit	11.2 ± 7.6 [*n* = 62]	11.2 ± 7.9 [*n* = 139]	0.977
Count of fibrinolysis	2 [1-3] [*n* = 62]	2 [1-3] [*n* = 136]	0.689
Fibrinolysis total (mg)	10.4 ± 4.3 [*n* = 60]	10.1 ± 4.4 [*n* = 132]	0.538
Ventilation (days)	4.1 ± 5.5 [*n* = 61]	3.9 ± 4.9 [*n* = 139]	0.786
Tracheotomy	11 (17.5%)	35 (24.8%)	0.268
Additional surgery	1 (1.6%)	6 (4.3%)	0.441
Intracranial pressure monitoring	13 (20.6%)	31 (22.0%)	0.879
External ventricular drain	10 (15.9%)	25 (17.7%)	0.713
Lumbar drain	3 (4.8%)	13 (9.2%)	0.400
Ventriculoperitoneal shunt	1 (1.6%)	9 (6.4%)	0.179
Mrs§ at discharge	4 [4-4]	4 [4-5] [*n* = 140]	0.120
Discharge disposition: rehabilitation clinic	53 (85.5%) [*n* = 62]	124 (87.9%)	0.649
Discharge disposition: home	3 (4.8%) [*n* = 62]	5 (3.5%)	0. 702
Discharge disposition: nursing home	1 (1.6%) [*n* = 62]	4 (2.8%)	1.000
Discharge disposition: further treatment	4 (6.5%) [*n* = 62]	7 (5.0%)	0.739
Discharge disposition: short time care	1 (1.6%) [*n* = 62]	1 (0.7%)	0.519

Data are mean with ± standard deviation, median [interquartile range], *n* (%), †Glasgow Coma Scale, ‡Intracerebral hemorrhage (ICH), §modified Rankin Scale (mRS)

Further baseline and treatment characteristics are summarized (shown in Table 1). Clinical data as well as discharge dispositions for participants [*n* = 63] and non-participants [*n* = 141] did not differ significantly (shown in Table 1).

### Follow-up parameters

Twenty-seven (42.9%) interviews were conducted with the affected person themself and 36 (57.1%) with a proxy. In detail, 19 interviews were conducted with spouses, 14 with children, and three with other relatives; 20 of them because the patients were not capable of independent participation, 16 because they were already deceased. Thus, 42.6% of the participants alive were represented. There were significant differences between the characteristics age (60.9 ± 10.1 years versus 71.4 ± 11.0 years), ICH score (2 [1-3] versus 3 [2.25-3]), and mRS score (3 [3-3] versus 4 [4-6]) at follow-up of self-participating, i.e. living patients (*n* = 27) and represented patients, i.e. living patients who are not capable of independent participation and already deceased patients (*p* = 36) (all *p* < 0.001). The mean follow-up period (time from ICH to interview) was 71.3 ± 39.7 months. The follow-up period of the deceased persons represented in the study (*n* = 16, 69.31.0 ± 41.09 months) did not differ significantly from that of the survivors (*n* = 47, 71.96 ± 39.617 months, *p* = 0.830).

### Sociodemographic data

Sociodemographic parameters are summarized (shown in Table 2).

**Table 2:** Sociodemographic data before and after the intracerebral hemorrhage

Sociodemographic data	Before ich*	After ich*
Married/partnered Single/widowed/divorced	48 (76.1%) 15 (23.9%)	49 (77.8%) 14 (22.3%)
At home Not at home [family/nursing home]	60 (95.2%) 3 (4.8%) [3/0]	47 (74.6%) 16 (25.4%) [2/14]
Employed [full time/part time] Not working [retired/unable] Housewife	19 (30.1%) [14/5] 41 (65.1%) [39/2] 3 (4.8%)	3 (4.8%) [2/1] 56 (88.9%) [47/9] 4 (6.3%)
Secondary school 13 (20.6%), apprenticeship 32 (50.8%), technical school 9 (14.3%), university or higher 9 (14.3%)		

Data are n (%), *Intracerebral hemorrhage (ICH)

The majority were married or partnered before and after ICH (both > 76.0%). Before ICH, many of the patients were already not working because they were already retired or were unable to work (65.1%). An increase of 23.8% in the rate of non-working persons was identified after ICH (the number of full time employees decreased from 14 to 2). The proportion of those living at home decreased by 20.6%. The highest level of education reported by most participants was apprenticeship, and 57 (90.5%) of the participants had children.

### Functional outcome (mRS)

**Fig. 1:**
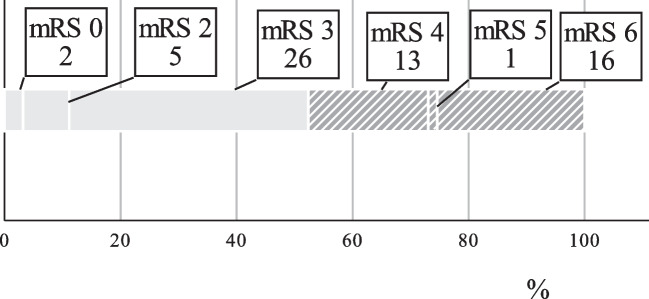
Functional outcome (mRS) at follow-up: mRS 0: 3.2%; mRS 1: 0%; mRS 2: 7.9%; mRS 3: 41.3%; mRS 4: 20.6%; mRS 5: 1.6%; mRS 6: 25.4%, *n* = 63

The median mRS score was three, good neurological outcome (mRS 0–3) was achieved in 52.4% (33/63) of the patients (shown in Fig. 1).

As expected, there was a significant association between higher ICH score at admission and lower functional outcome at follow-up (*r* = 0.514, *p* < 0.001).

### Health-related quality of life (hrQoL)

The average QOLIBRI-OS index score was 49.55 ± 27.75 with a maximum achievable score of 100 (shown in Table 3).

**Table 3:** QOLIBRI-OS items and scores of all participants, of patients and proxies, of retrospectively consenting participants (ReCoPa) and retrospectively non-consenting participants (ReNonCoPa)

QOLIBRI-OS	All [*n* = 62]	Patient [*n* = 27]	Proxy [*n* = 35]	*p*-value	ReCoPa* [*n* = 51]	ReNonCoPa† [*n* = 6]	*p*-value
Index	49.55 ± 27.75	63.71 ± 19.69	38.63 ± 28.34	**0.001**	54.97 ± 25.11	13.90 ± 26.72	**0.004**
Physical condition	2.71 ± 1.27	3.30 ± 1.20	2.26 ± 1.15	**0.001**	2.96 ± 1.22	1.33 ± 0.82	**0.005**
Cognition	3.11 ± 1.34	3.81 ± 1.11	2.57 ± 1.27	**< 0.001**	3.31 ± 1.26	1.17 ± 0.41	**< 0.001**
Emotion	3.18 ± 1.31	3.59 ± 1.08	2.86 ± 1.40	**0.043**	3.39 ± 1.22	1.67 ± 1.21	**0.007**
Daily life	2.61 ± 1.30	3.30 ± 0.91	2.09 ± 1.31	**< 0.001**	2.84 ± 1.24	1.50 ± 1.23	**0.019**
Social relationships	3.47 ± 1.34	3.81 ± 1.00	3.20 ± 1.51	0.135	3.63 ± 1.17	2.17 ± 1.84	**0.042**
Future prospects	2.81 ± 1.32	3.48 ± 0.97	2.29 ± 1.32	**0.001**	3.06 ± 1.24	1.50 ± 1.23	**0.014**

Data are mean with ± standard deviation, *retrospectively consenting participants (ReCoPa), †retrospectively non-consenting participants (ReNonCoPa)

**Fig. 2:**
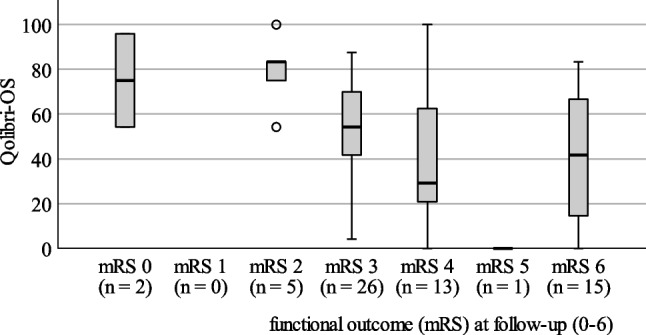
QOLIBRI-OS according to functional outcome (mRS) at follow-up, *n* = 62

No significant difference in hrQoL scores was found between male (45.27 ± 28.17) and female (55.10 ± 26.68, *p* = 0.279), age did not play a role (r = -0.234, *p* = 0.067), nor did the length of the follow-up period (r = 0.071, *p* = 0.583). Within the individual items of the QOLIBRI-OS with scores from zero to five (shown in Table 3), individual satisfaction was significantly higher for “emotions”, “social relationships”, and “cognition” than for “physical condition” (*p* < 0.001, *p* < 0.001, *p* = 0.007) and “daily life (*p* < 0.001, *p* < 0.001, *p* = 0.001). Additionally, satisfaction was also rated significantly higher for “emotions” and “relationships” than for “future prospects” (*p* = 0.002, *p* < 0.001). Interestingly, there was no correlation between lower hrQoL and higher ICH score (r = -0.148; *p* = 0.249) or between hrQoL and functional status (mRS) at discharge (r = -0.158; *p* = 0.219). But hrQoL was correlated significantly negatively with the functional status (mRS) at the time of follow-up (r = -0.381, *p* = 0.002; *n* = 62). This analysis is based on the correlation of the mean total values of the QOLIBRI-OS and the ordinal mRS (shown in Fig. 2).

Patients rated their hrQoL significantly higher than proxies did (*p* = 0.001). Likewise, the items “physical condition”, “cognition”, “daily life”, “future prospects” and “emotions”, were rated significantly higher. The item “relationships” was rated higher but without significant difference (shown in Table 3).

### Retrospective consent

Fiftyeight of 63 participants were able to answer the question concerning retrospective consent, which were included in the following analysis. All five participants who did not answer this question were proxies representing patients with a mRs score of four or six. Overall, 52 (89.7%) of the patients would retrospectively approve of the intervention, while six (10.3%) would retrospectively disapprove. No significant relationship between a higher ICH score and retrospective consent (ICH score 0: 100% [1/1]; 1: 88,9% [8/9]; 2: 100% [19/19]; 3: 79,2% [19/24]; 4: 100% [5/5]) could be determined (*p* = 0.323). Patients who left the hospital with a good functional status (mRS 0–3) all gave their retrospective consent (8/8). Those who left the hospital with a poor functional status (mRS 4–5) gave their retrospective consent in 88% (mRS four: 94.7% [36/38]; mRS five: 66.7% [8/12]). Of the self-respondents, 100% agreed to the retrospective consent (27/27). If the question was answered by a proxy, a total of 80.7% retrospectively consented (25/31). In detail, four spouses, one child, and one relative did not agree to the retrospective consent. There is no significant difference between the retrospective consent rate for patients treated in the first 5 years [January 2010-May 2015] (*n* = 32, yes = 29, no = 3) compared with the last 5 years [June 2015-October 2020] (*n* = 26, yes = 23, no = 3, *p* = 1.000). The will to retrospectively consent to the treatment was significantly related to the subcohorts “living at home” after ICH and “independent interview participation” (shown in Table 4).

**Table 4:** Association between retrospective consent (yes/no) and sociodemographic data

Items [*n* = 58]	Yes [*n* = 52]	No [*n* = 6]	*p*-value
Female Male	25 (92.6%) 27 (87.1%)	2 ( 7.4%) 4 (12.9%)	0.675
Age	66.8 ± 11.6	60.5 ± 12.1	0.211
Married/partnered Single/divorced/widowed	40 (88.9%) 12 (92.3%)	5 (11.1%) 1 ( 7.7%)	1.000
Home Not at home	44 (95.7%) 8 (66.7%)	2 ( 4.3%) 4 (33.3%)	**0.014**
Education	Apprenticeship	Apprenticeship	0.433
Job No job	3 (100%) 49 (89.1%)	0 ( 0.0%) 6 (10.9%)	1.000
Children No children	47 (90.4%) 5 (83.3%)	5 ( 9.6%) 1 (16.7%)	0.497
Patient Proxy	27 (100%) 25 (80.6%)	0 ( 0.0%) 6 (19.4%)	**0.026**
Follow-up-periode	69.4 ± 39.5	78.3 ± 39.0	0.664

Data are mean with ± standard deviation and n (%)

Both of the mentioned subcohorts had significantly better functional status at follow-up (living at home: mRS 3 [3-4] versus living not at home: mRS 4 [4-6], *p* = 0.003; independent responders: mRS 3 [3-3] versus proxy: 4 [4-6], *p* < 0.001).

**Fig. 3:**
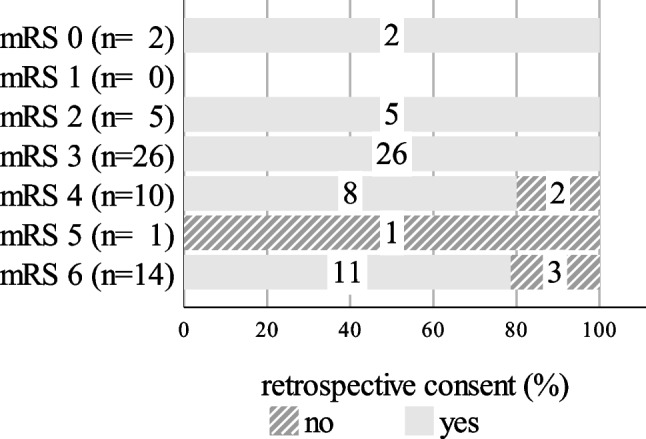
Retrospective consent according to functional outcome (mRS) at follow-up: mRS 0: 2/2 [100.0%]; mRS 1: 0/0; mRS 2: 5/5 [100.0%]; mRS 3: 26/26 [100.0%]; mRS 4: 8/10 [80.0%]; mRS 5: 0/1 [0.0%]; mRS 6: 11/14 [78.6%], *n* = 58

All 33 patients who had a good functional outcome (mRS 0–3) at follow-up agreed retrospectively. A high rate of 76.0% (19/25) would also have given their consent despite poor outcome (shown in Fig. 3).

Overall, retrospectively consenting participants (ReCoPa) had a significantly better long-term functional outcome (mRS 3 [3-4]) than retrospectively non-consenting participants (ReNonCoPa) (mRS 5.5 [4.25-6], *p* = 0.008). Retrospective consent is significantly related to the achievement of a good functional outcome (*p* = 0.004, *n* = 58).

**Fig. 4:**
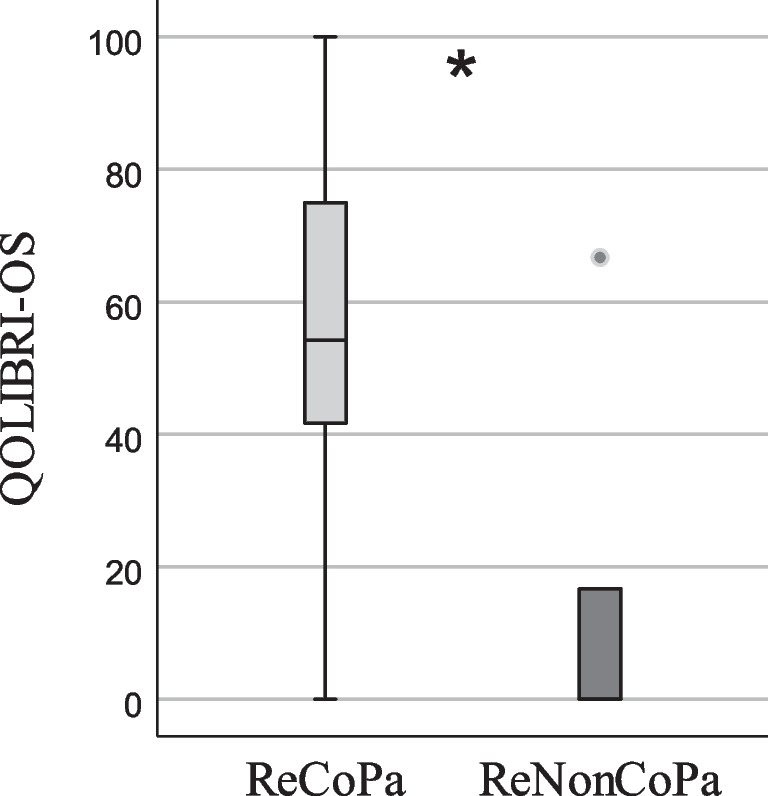
QOLIBRI-OS indexes of retrospectively consenting participants (ReCoPa) and retrospectively non-consenting participants (ReNonCoPa), *n* = 57

**Fig. 5:**
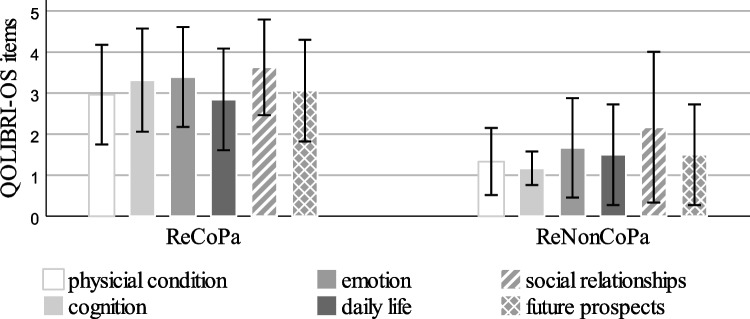
QOLIBRI-OS items (physical condition, cognition, emotion, daily life, social relationships, future prospects) of retrospectively consenting participants (ReCoPa) and retrospectively non-consenting participants (ReNonCoPa), *n* = 57

Moreover, ReCoPa had a significantly higher QOLIBRI-OS index score as well as significantly higher scores in every single QOLIBRI-OS item (shown in Table 3; Figs. 4 and 5). The two ReCoPa with a QOLIBRI-OS-score of 0 were proxies.

## Discussion

In potentially devastating conditions like ICH, (severe) neurological impairment frequently occurs as well as the question if a certain therapeutical approach is justified. In the literature, functional outcome is often used as an argument pro or against therapy. The use of patient-reported outcome measures is mostly excluded from these discussions. However, our data show that explicitly these outcomes measures require comprehensive consideration.

When comparing our population with a population that included mainly patients with ischemic stroke (93%), our results indicate that hemorrhagic stroke, although less frequent than ischemic stroke, possibly causes greater limitations in hrQoL [20]. Therefore, similar attention should be paid to research on hemorrhagic stroke.

Our data demonstrate that hrQoL after minimally invasive thrombolysis therapy for ICH does not depend on the initial severity of bleeding (ICH score). More severe ICH cases should therefore not automatically declassify for surgical therapy. Excluded from this finding are patients with an ICH score of five and six, as they are not part of the follow-up cohort due to a mortality rate of 100%.

Even though the functional outcome appears to have a major impact on the hrQoL, which is consistent with other studies, the mRS should not be used as a synonymous construct for hrQoL. HrQoL has been shown to be a multidimensional construct that is influenced by far more aspects than just the functional component [21, 22]. As neurological outcome after ICH is often objectively measured as poor but reflects only one dimension of the resulting circumstances, focussing on this parameter would represent a massively attenuated treatment benefit by ignoring potential positive factors [23].

Another main result of our data was that minimally invasive ICH therapy seemed acceptable from the patients’ view. This conclusion can be derived from the high retrospective consent rate. Therefore, it would be fatal to omit an operation that reduces blood volume effectively and decreases mortality rate on the one hand, and additionally has a high likelihood of providing a condition judged acceptable to the individual [5, 24–27]. Honeybul et al. however raise ethical concerns about whether retrospective consent can truly justify therapy because of possibly impaired cognitive skills after stroke and the emotional influence of such a question [28]. However, the apprehension that surgical therapy for ICH may convert mortality into survival with unacceptable outcome should be disproved because of the high rate of consent and consequently a high acceptance of the resulting circumstances.

Regarding the dilemma that decision making for patients after initially severe bleeding involves great uncertainty, it is encouraging that our data show that many of these individuals accept the resulting circumstances. Consequently, individual acceptance does not seem to be predictable in the initial situation. Thus, decision making should not be based on ICH score and expected functional outcome solely.

In case of the significantly lower agreement rate of proxies, a potential observer bias should be considered when interpreting the results [29]. However, especially in case of a disease like stroke, which is often associated with disability and functional impairment, the inclusion of proxies prevents the systematic exclusion of severely affected individuals. Thus, the proxy survey should be considered as a strength in terms of information gain, representative mapping, and increased generalizability [19]. A possible explanation of this bias could be the disability paradox and the focus illusion [30, 31]. This means that outsiders tend to focus on the changed circumstances (e.g. disability), so that unchanged factors such as social relationships, emotions, and the circumstance of survival are attributed less importance [23, 32, 33]. People with a disability are often reduced to the visible limitations without considering that due to non-visible values, they can still have a satisfying life [30]. Nevertheless, the lower rate of consent by proxy could also be due to the stressful situation arising from the impairments or because of the significantly worse functional status of the represented patients [34].

Contextual factors, reflecting independence like living in one’s own home, and communication skills should be optimized in the clinical course since they seem to modify the acceptance of impaired life circumstances [35].

The potential benefits of surgery were discussed with every patient, in the sense that surgery might represent a relieve of a focal neurological deficit. However also the possibility of negative outcome or death was iterated. It might be true that facing death is a strong argument in favor of surgery. However, the risk of survival with possibly intolerable disabilities could be an argument against surgery. This controversy has been a reason for us to ask for retrospective consent. From the literature we know, that there often is no linear relationship between hrqol and functional outcome. Frequently, also patients with neurological diseases report a satisfactory to high hrqol associated with low functional outcome [30]. With the high rate of retrospective consent even in cases with poor outcome, minimally invasive ICH therapy represents a clear therapeutic success. We think that the suggestion made by Christensen et al. to use the mRS to measure hrQoL should be avoided, as this would indicate that death is worse than survival with an mRs score of five, which seems to be refuted by our data and also in literature [21, 31, 36]. Moreover, treatment success cannot be dichotomized into “survival” and “death” because “survival” is not considered the same by all [37].

The disease-specific hrQoL seems to represent the subjective acceptance of the resulting life circumstances precisely and in contrast to functional outcome analyses, by considering all aspects of life [8, 10, 38]. Consequently, it can be used as a criterion for success of rtPA lysis therapy in ICH.

### Limitations

Limitations can be found in the relatively small follow-up data set because of missing standard post-event linkage. Failure to collect contact information, conduct patient education, and obtain consent while the patients were still in the hospital, made contact more difficult, increased the burden on the participants, and thus led to a higher lost-to-follow-up rate. Thus, a valid representation of all potential participants cannot be assumed [39]. The small number of retrospectively non-consenting participants (ReNonCoPas) did not allow a multivariate analysis, and consequently the detection of influential variables on retrospective consent. The variable follow-up period implies heterogeneity within the own study collective. The lack of a randomized comparison group may be considered a limitation, as the separate consideration dependent on the chosen treatment modality is a central element in current neurosurgical outcome research [5]. Moreover, the proxy interviews could account for bias, as the assessment of hrQoL by observers may differ from the self-report of the affected person [19, 30]. Since the willingness to participate was greater among the affected persons or the proxies of the ones who were still alive than among the proxies of those who had already died, an underrepresentation of severely affected persons is evident. Also, the interviews took place during the COVID-19 pandemic, which might have had an influence on the evaluation [40].

## Conclusion

All in all, this paper was able to capture the subjective perspective on hrQoL with resulting life circumstances after ICH. This seems to contradict the clinically determined neurological status. Treatment is justified even when baseline variables lead to an assumption of a high probability of poor neurological outcome. Functional outcome analyses should no longer be used for therapy justification. The establishment of patient-centred evaluations should find a broader clinical implementation serving as an outcome criterion for success of rtPa lysis therapy in ICH.

## Data Availability

No datasets were generated or analysed during the current study.
